# Identification of benzoxazine scaffold derivatives as novel CDK4 inhibitors for the treatment of cancer

**DOI:** 10.1080/14756366.2026.2681363

**Published:** 2026-09-30

**Authors:** Chao Lin, Shaofeng Sun, Huateng Zhang, Dan Yang, Youyan Wei, Bo Li

**Affiliations:** a Department Traditional Chinese Medicine, Shandong College of Traditional Chinese Medicine, Yantai, Shandong, China; b Yantai Key Laboratory of Nanomedicine and Advanced Preparations, Yantai Institute of Materia Medica, Yantai, Shandong, China; c Shandong Luye Pharmaceutical Co. Ltd, Yantai, China

**Keywords:** CDK4, benzoxazine, cancer

## Abstract

Cyclin-dependent kinase 4, a pivotal regulator of cell cycle progression, is widely recognised as a therapeutic target for controlling tumour proliferation. Inspired by the reported CDK4 inhibitor PF-07220060, a series of novel benzoxazine derivatives were designed, synthesised, and evaluated for their biological activity. From this series, compound **8j** emerged as the most potent anti-proliferative agent. Subsequent *in vitro* studies showed **8j** effectively inhibits HCT116 cell proliferation, clonogenic survival, and migration. Mechanistic investigations revealed that **8j** induces G1 phase cell cycle arrest and promotes apoptosis, which was corroborated by the observed concentration-dependent downregulation of key cell cycle regulators, including CDK4 and cyclin D1. Molecular docking and dynamics simulations substantiated the binding mode of **8j**within the ATP-binding pocket of CDK4, revealing stable interactions characterised by key hydrogen bonds and hydrophobic contacts. Collectively, these results identify compound **8j** as a promising lead candidate worthy of further structural optimisation and preclinical development.

## Graphical abstract

**Figure:**
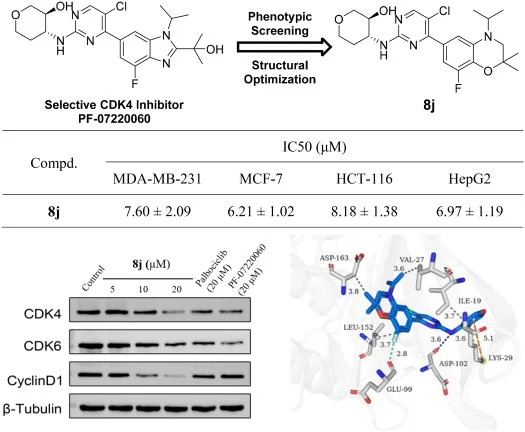

## 1. Introduction

Cancer remains a leading cause of morbidity and mortality worldwide, with approximately 18.1 million new cases and 9.5 million deaths reported annually, which highlights the critical demand for novel therapeutic strategies^1^. A fundamental hallmark of oncogenesis is the loss of the dysregulated control of the cell cycle, leading to uncontrolled proliferation and apoptotic evasion^2–5^. The cyclin-dependent kinase family, comprising serine/threonine kinases, serves as a central coordinator of cell cycle progression, transcriptional regulation, and other essential cellular functions^6–8^. The G1-to-S phase transition is primarily facilitated by CDK4 and CDK6, which, upon forming complexes with D-type cyclins, mediate phosphorylation of the retinoblastoma protein^9–11^. The clinical efficacy of first-generation CDK4/6 inhibitors (palbociclib, ribociclib, and abemaciclib) (Figure 1) has established CDK4/6 inhibition as a validated therapeutic approach^12–15^. However, these agents are associated with dose-limiting toxicities, including neutropenia, anaemia, and gastrointestinal disturbances^16^^,^^17^, largely due to concurrent CDK6 inhibition and its role in haematopoiesis^18–20^. Consequently, developing selective CDK4 inhibitors with improved safety profiles remains an urgent unmet need^21–23^.

**Figure 1.**
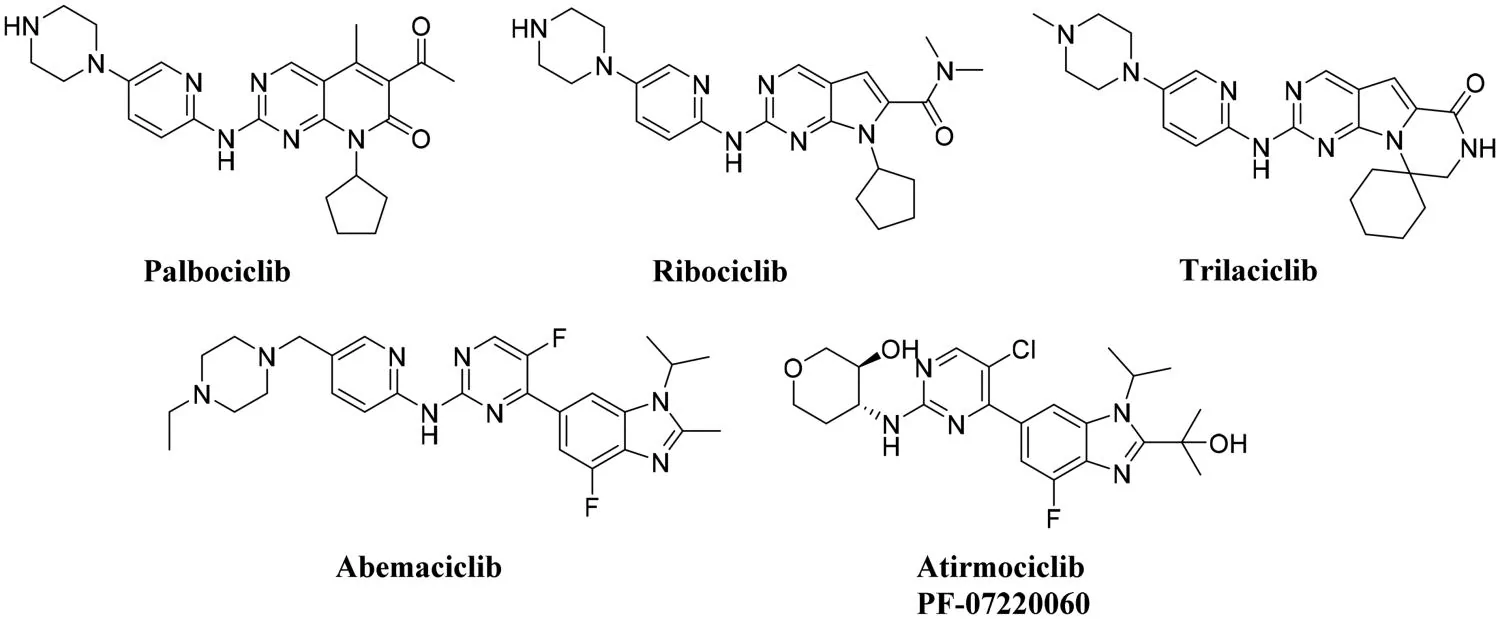
Chemical structures of selected representative CDK4/6 inhibitors.

Recent efforts have focused on optimising CDK4 selectivity over CDK6 to mitigate off-target effects. The aminopyrimidine-based inhibitor PF-07220060 (atirmociclib) exemplifies this approach, demonstrating high CDK4 potency (IC_50_ = 0.53 nM) and 26-fold selectivity over CDK6^24^. Structural analyses reveal that selectivity arises from differential flexibility in the G-loop of CDK4 versus CDK6, allowing preferential binding of inhibitors with specific steric and electronic properties. Nevertheless, therapeutic resistance remains a major challenge in clinical oncology^25^. Several mechanisms contributing to this resistance have been identified, including molecular alterations in the CDK4/6/cyclin D axis or its key downstream target, retinoblastoma protein, compensatory activation of other CDK complexes, and enhanced signalling through upstream pathways, particularly the PI3K/mTOR cascade^26–28^. Consequently, there is a clear need to design and develop novel CDK4 inhibitors based on new chemical scaffolds.

Our drug design strategy was initiated with the clinically relevant CDK4 inhibitor PF-07220060 as the lead structure. To explore novel chemical space and potentially improve upon its profile, we employed a scaffold-hopping approach, replacing benzimidazole with a benzoxazine moiety. This modification was rationally guided by preliminary molecular docking studies, which suggested that the benzoxazine scaffold could be effectively accommodated within the ATP-binding pocket of CDK4, potentially engaging in key hinge-region interactions similar to PF-07220060. We hypothesised that strategic substitutions on the benzoxazine core would allow us to modulate potency, selectivity, and physicochemical properties. A focused library of derivatives was subsequently synthesised to probe the structure–activity relationship at these positions. From this series, compound **8j** emerged as the most potent anti-proliferative agent in preliminary screening. It was therefore selected for comprehensive mechanistic evaluation, which confirmed its role as a promising CDK4 inhibitor. Collectively, these findings suggest that compound **8j**provides a promising structural foundation for developing next-generation CDK4 inhibitors and merits further in-depth investigation.

## 2. Results and discussion

### 2.1. Chemical synthesis of benzoxazine derivatives

The target benzoxazine derivatives **8a–8n** were synthesised following the route depicted in Scheme 1. The synthesis was initiated from commercially available starting materials: substituted 2-amino-4-bromophenol **1** and chloroacetyl chloride **2**. Initially, cyclisation of compounds **1** with a series of substituted chloroacetyl chlorides **2** afforded intermediates **3**. These intermediates were then reduced with borane to yield benzoxazine scaffold derivatives **4**. Subsequently, in the presence of sodium hydride (NaH), derivatives **4** reacted with alkyl halides in dry dimethylformamide (DMF) to provide intermediates **5**. Intermediates **5** underwent a Miyaura borylation reaction to form boronic ester intermediates **6**. Next, a Suzuki coupling reaction between intermediates **6** and 2,4,5-trichloropyrimidine yielded intermediates **7**. Finally, intermediates **7** were substituted with (3S,4R)-4-aminooxan −3-ol hydrochloride, which was obtained from a commercial source, to afford the target compounds **8a**–**8n** in moderate to good yields (52–69%). The structures of all final products were characterised by ^1^H/^13^C NMR and high-resolution mass spectrometry (HRMS).

**Scheme 1.**
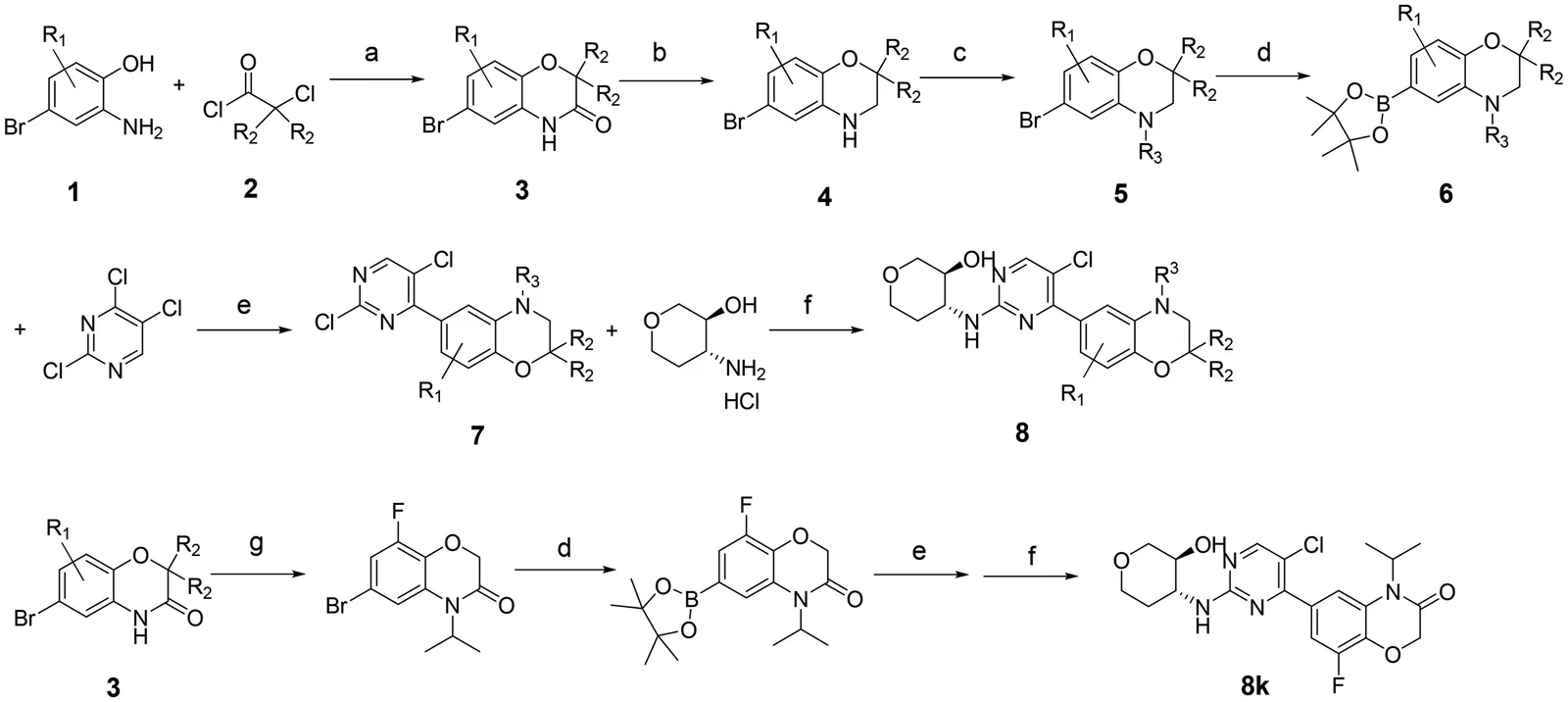
Synthetic route for the preparation of target compounds **8a**–**8n**.

Reagents and conditions: (a) NaHCO_3_, 1,2-Dimethoxyethane, r.t.; Cs_2_CO_3_, 80 °C, 1 h; (b) Borane-tetrahydrofuran complex, THF, reflux, 2.5 h; (c) NaH, dry DMF r.t., 4 h; (d) Pd(dppf)Cl_2_, CH_3_COOK, dioxane, N_2_, 90 °C, 6 h; (e) Pd(PPh_3_)_4_, Na_2_CO_3_, dioxane, H_2_O, N_2_, 90 °C, 4 h; (f) DIPEA, CH_3_CN, 80 °C, 48 h.

### 2.2. *In vitro* antiproliferative evaluation and structure–activity relationship (SAR)

A series of newly synthesised target compounds (**8a**–**8n**) were evaluated for their antiproliferative activities against four cancer cell lines: HCT-116 (colon cancer), MDA-MB-231 (breast), MCF-7(breast), and HepG2 (hepatoma) using the MTT assay. The known CDK4/6 inhibitors Palbociclib and PF-07220060 were used as positive controls. The *in vitro* antiproliferative activities of compounds **8a–8n** are summarised in Table 1. The IC_50_ values indicate that the potency of these compounds is highly dependent on both their structural features. Initial structure–activity relationship studies focused on the substituents at the nitrogen atom of the benzoxazine scaffold, which were modified with different alkyl and acyl groups. Most alkyl-substituted compounds demonstrated significant antiproliferative activity against tumour cells, with compound **8b** exhibiting potent activity across all four tested tumour cell lines. In contrast, compounds with acyl substitutions showed markedly reduced antiproliferative efficacy. Furthermore, introduction of substituents at the 2- or 3-position of the morpholine ring was investigated. Cytotoxicity evaluation revealed that dimethyl substitution at the 3-position of the morpholine ring significantly enhanced antitumor activity, yielding compound **8j**, which proved to be the most potent among all target compounds. Finally, to probe the influence of electronic effects, various substituents were introduced onto the benzene ring of the benzoxazine core. Comparison of the cellular activities of compounds **8l–8m** indicated that electron-withdrawing groups on the benzene ring are beneficial for antitumor activity. Additionally, an *in vitro* cytotoxicity assessment of compound **8j** against the human normal hepatocyte cell line L02 was performed. As presented in Table 2, similar to the Palbociclib and PF-07220060, compound **8j** demonstrated low toxicity towards L02 cells, with an IC_50_ value greater than 50 µM.

**Table 1.** Determination of IC_50_ values for the synthesised compounds against cancer cell lines.

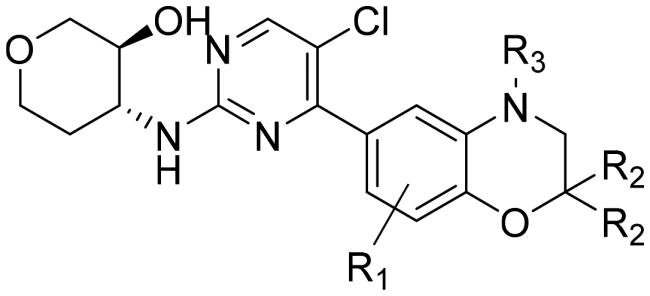							
Compd.	R_1_	R_2_	R_3_	IC_50_^a^ (μM)			
				MDA-MB-231	MCF-7	HCT-116	HepG2
8a	8-F	H	-CH_3_	7.04 ± 0.72	18.41 ± 3.09	17.97 ± 1.24	6.68 ± 0.71
8b	8-F	H	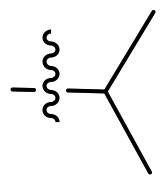	12.31 ± 1.55	10.38 ± 2.12	15.91 ± 1.05	8.34 ± 0.52
8c	8-F	H	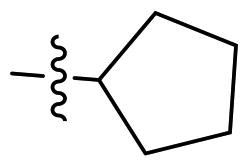	ND^c^	ND	>30	ND
8d	8-F	H	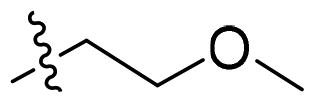	8.28 ± 1.80	11.55 ± 0.53	19.44 ± 1.37	10.06 ± 2.21
8e	8-F	H	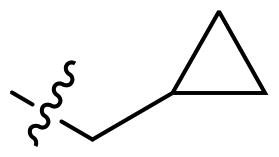	6.55 ± 0.30	9.38 ± 0.82	12.28 ± 1.06	9.87 ± 0.70
8f	8-F	H	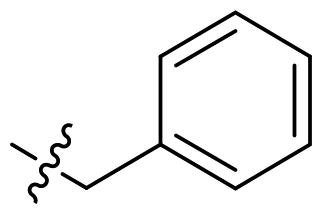	ND	ND	>30	ND
8g	8-F	H	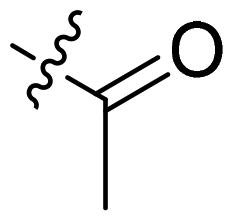	ND	ND	>30	ND
8h	8-F	H	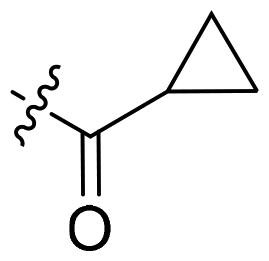	ND	ND	>30	ND
8i	8-F	H	H	8.75 ± 0.57	10.67 ± 0.85	16.90 ± 1.72	12.77 ± 0.54
**8j**	8-F	–CH_3_	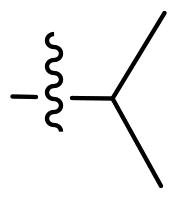	7.60 ± 2.09	6.21 ± 1.02	8.18 ± 1.38	6.97 ± 1.19
8k	8-F	H	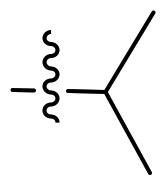	9.76 ± 1.05	9.99 ± 1.37	24.97 ± 1.68	12.56 ± 0.82
8 l	H	H	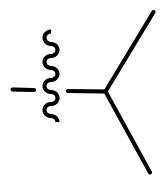	ND	ND	>30	ND
8m	8-OCH_3_	H	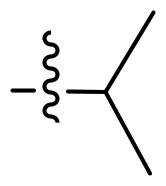	ND	ND	>30	ND
8n	7-F	H	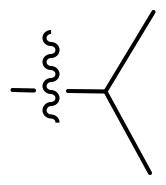	9.55 ± 0.94	8.00 ± 0.51	14.61 ± 1.11	9.20 ± 0.87
Palbociclib^b^	–			7.45 ± 1.81	6.48 ± 0.38	19.72 ± 2.42	10.20 ± 3.66
PF-07220060^b^	–			10.08 ± 2.21	7.47 ± 1.17	19.49 ± 2.53	7.38 ± 1.04

^a^The IC_50_ values are reported as the mean ± standard deviation (SD) derived from three independent experiments.

^b^Palbociclib and PF-07220060 served as the positive control.

^c^ND = not determined.

**Table 2.** IC_50_ values of compounds **8j**, Palbociclib and PF-07220060 against human normal hepatocyte L02.

Compd	IC_50_^a^ (μM)
**8j**	>50
Palbociclib^b^	>50
PF-07220060^b^	>50

^a^The IC_50_ values are reported as the mean ± standard deviation (SD) derived from three independent experiments.

^b^Palbociclib and PF-07220060 served as the positive control.

### 2.3. Molecular docking analysis and molecular dynamics simulation

Molecular docking simulations were employed to elucidate the binding mode of **8j** within the ATP-binding pocket of CDK4 kinase. As shown in Figure 2, **8j** exhibited a high binding affinity with a docking score of −8.9 kcal/mol. The binding pose of **8j** (blue) demonstrated significant spatial overlap with the reference ligand PF-07220060 (yellow), indicating a conserved pharmacophore orientation. Specifically, **8j** formed a critical hydrogen bond with the hinge residue Asp102 (3.60 Å), a hallmark of potent kinase inhibition. The ligand scaffold was further stabilised by a robust hydrophobic interface involving Lys29 (3.58 Å), Val27 (3.60 Å), Ile19 (3.69 Å), Leu152 (3.73 Å), and Asp163 (3.80 Å). Additionally, a π-cation interaction was observed between the aromatic ring of **8j** and Lys29 (5.08 Å). Notably, the fluorine atom of **8j** formed a typical halogen bond with Glu99 (distance: 2.79 Å, angle: 173.72°), providing high directionality and binding specificity.

**Figure 2.**
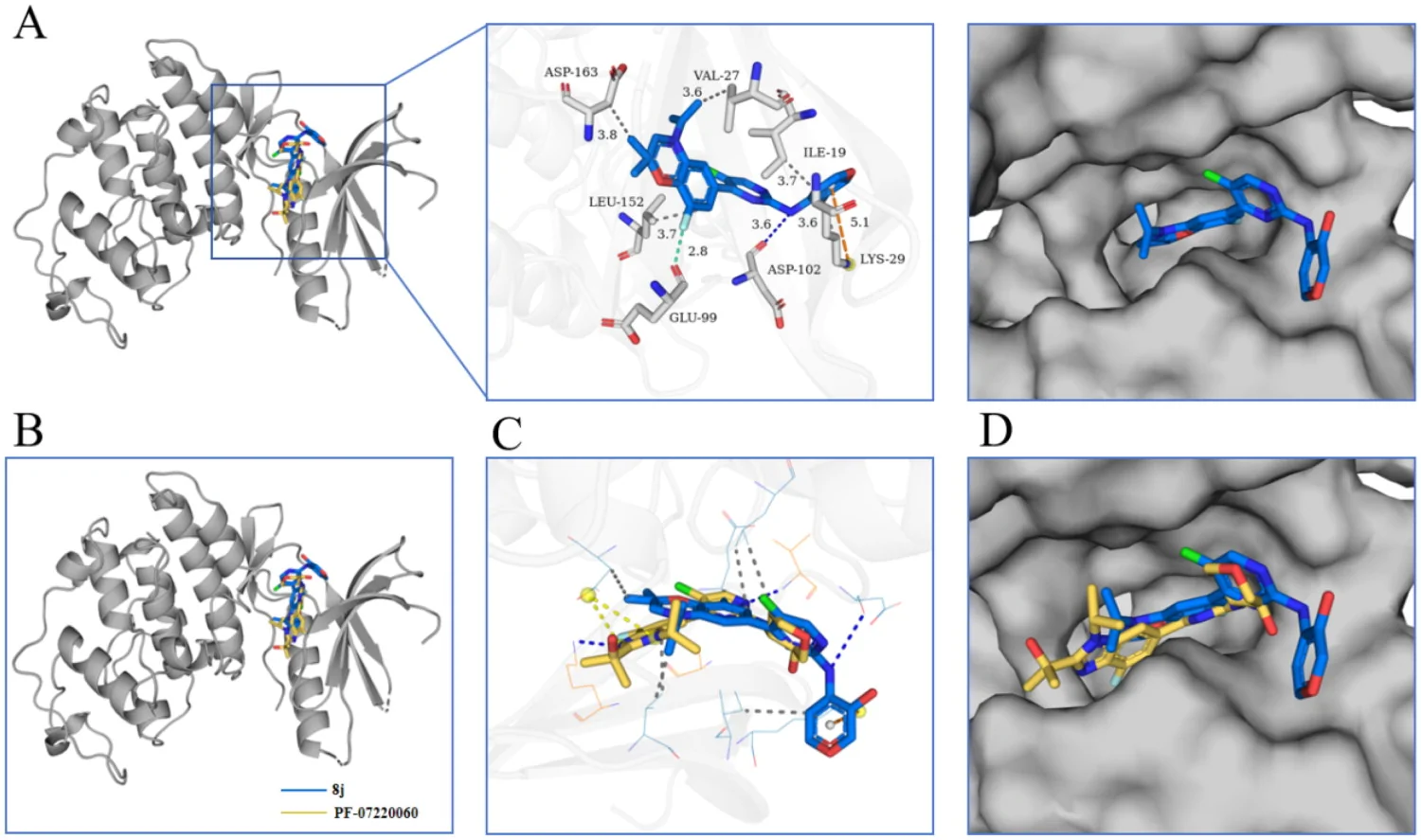
Molecular docking simulation and binding mode analysis of compound **8j** at the CDK4 kinase active site. The crystal structure of CDK4 (grey ribbon representation, PDB code: 9PE8) was used for docking. Compound **8j** (blue stick model) and the PF-07220060 (yellow stick model) are compared. (A) Detailed view of the interactions between **8j** and key residues of CDK4. In the graphical representation, hydrogen bonds are indicated by green dashed lines, π-cation interactions by orange dashed lines, halogen bonds by cyan dashed lines and hydrophobic contacts by grey dashed lines. (B, C) Superposition of **8j** and PF-07220060 within the ATP-binding pocket, showing highly overlapping spatial orientations. (D) Surface representation of the CDK4 binding pocket illustrating the structural complementarity with **8j**. Key residues including Asp102, Lys29, and Glu99 are labelled.

To evaluate the dynamic stability of the CDK4–**8j** complex, a 100 ns MD simulation was conducted (Figure 3). The RMSD of the protein backbone stabilised within 0.20–0.30 nm, while the ligand maintained a remarkably low RMSD of ∼0.08 nm, indicating a highly stable binding state (Figure 3A,B). The structural integrity was further supported by the stable fluctuations of SASA (135–155 nm^2^) and the radius of gyration (Rg, 1.925–2.000 nm), suggesting that the protein remained compactly folded throughout the simulation (Figure 3C,D). RMSF analysis confirmed that residues in the binding pocket exhibited low flexibility (<0.3 nm), ensuring a rigid environment for ligand anchoring (Figure 3E). A consistent number of intermolecular hydrogen bonds (average = 1.0) were maintained, providing a continuous stabilising force (Figure 3F). The FEL analysis revealed a single, deep energy minimum basin, confirming that the complex attained a thermodynamically favoured conformation (Figure 3G–H). Representative snapshots from 0 to 100 ns illustrated that **8j** remained firmly anchored within the binding pocket without significant displacement (Figure 3I), corroborating the stability and high affinity of the CDK4–**8j** interaction.

**Figure 3.**
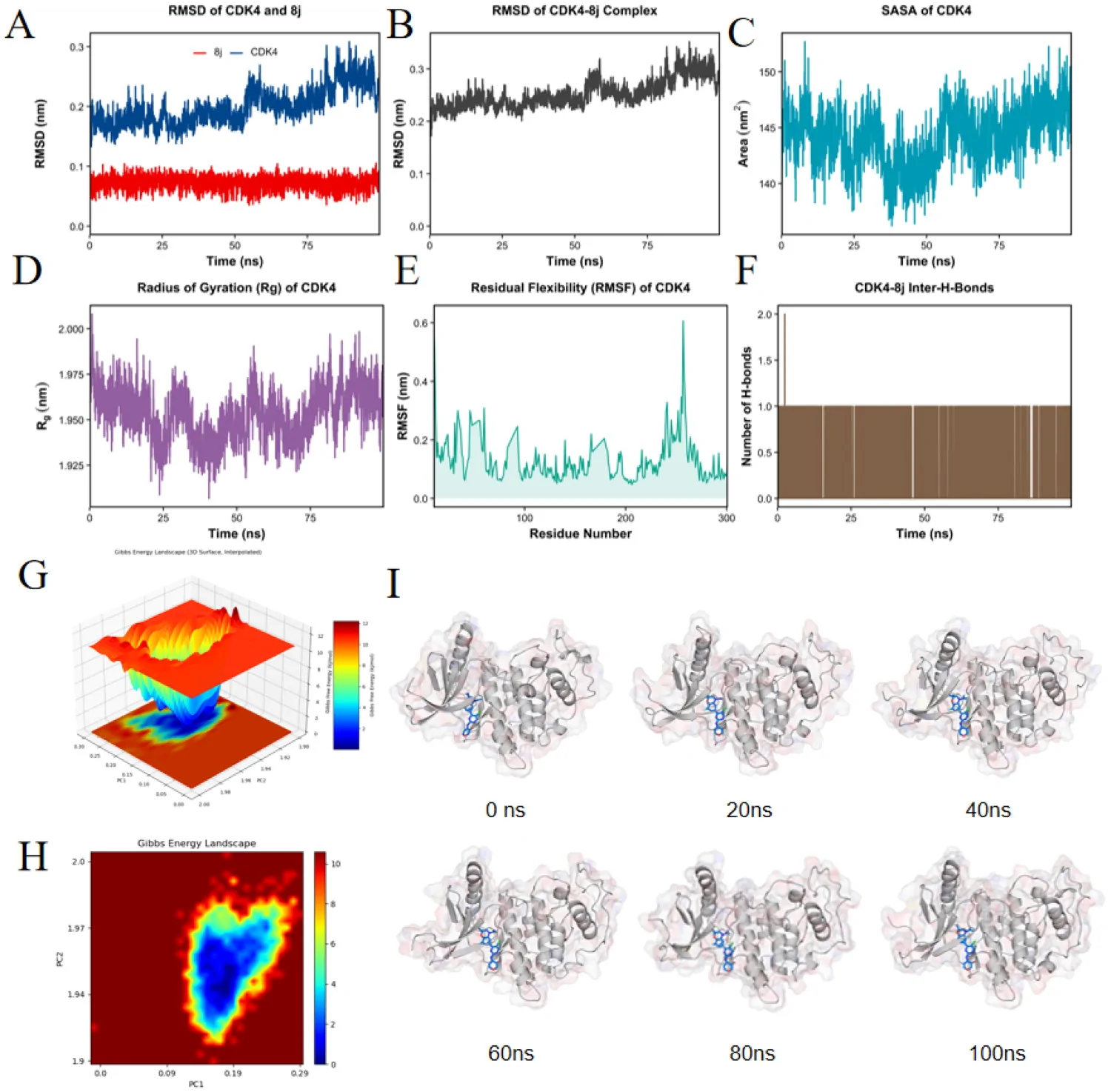
Trajectory analysis and stability assessment of the CDK4–**8j** complex during 100 ns molecular dynamics simulation. (A) Root-mean-square deviation of the CDK4 protein backbone (blue) and ligand **8j** (red). (B) RMSD of the protein-ligand complex. (C) Solvent accessible surface area of the complex. (D) Radius of gyration (Rg) of the CDK4 protein during the 100 ns trajectory. (E) Root-mean-square fluctuation (RMSF) of individual CDK4 residues. (F) Evolution of the number of hydrogen bonds formed between **8j** and CDK4 over time. (G, H) Gibbs free energy landscape (FEL) and projection of the first two principal components derived from principal component analysis. (I) Time-resolved snapshots of the **8j**-CDK4 binding mode, showing the persistent anchoring of the ligand in the ATP-binding pocket.

### 2.4. Inhibitory effect of compound 8j on the clonogenic survival of HCT116 cells

The long-term antiproliferative activity of **8j** was evaluated using a clonogenic assay. As depicted in Figure 4, treatment with **8j** led to a significant, dose-dependent reduction in colony formation. Collectively, these findings indicate that compound **8j** effectively compromises the clonogenic potential and sustained proliferative capacity of HCT116 cells.

**Figure 4.**
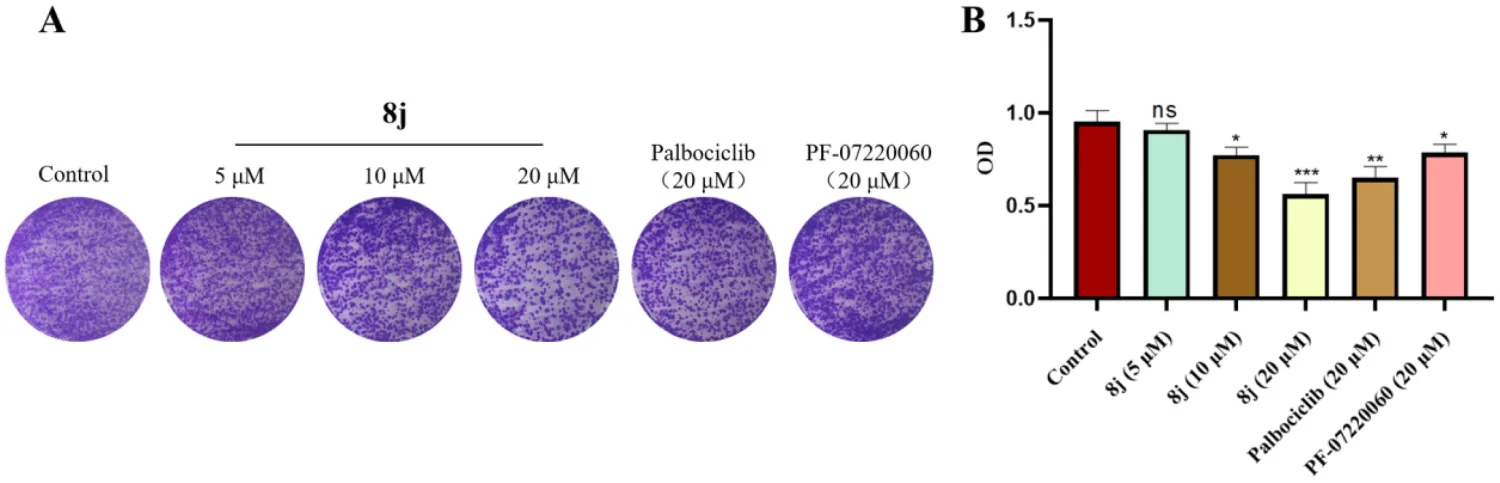
Inhibitory effect of compound **8j** on the clonogenic survival of HCT116 cells.(A) Representative images of **8j** on clonal formation of HCT116 cells. (B) Quantitative analysis of colony formation. *P < 0.05, **P < 0.01, ***P < 0.001 compared with control.

### 2.5. Effects of 8j on HCT116 migration ability

Cell migration represents a pivotal process in tumour progression and metastatic dissemination. The anti-migratory activity of **8j** was evaluated using a scratch wound assay. As shown in Figure 5, untreated HCT116 cells exhibited robust migratory capacity, leading to progressive wound closure. In contrast, treatment with increasing concentrations of **8j** led to a marked, dose-dependent inhibition of cell migration, as evidenced by a significant reduction in the rate of wound closure. These results demonstrate that compound **8j** produces a significant and quantifiable suppression of HCT116 cell migration, exhibiting clear concentration and time-dependent characteristics.

**Figure 5.**
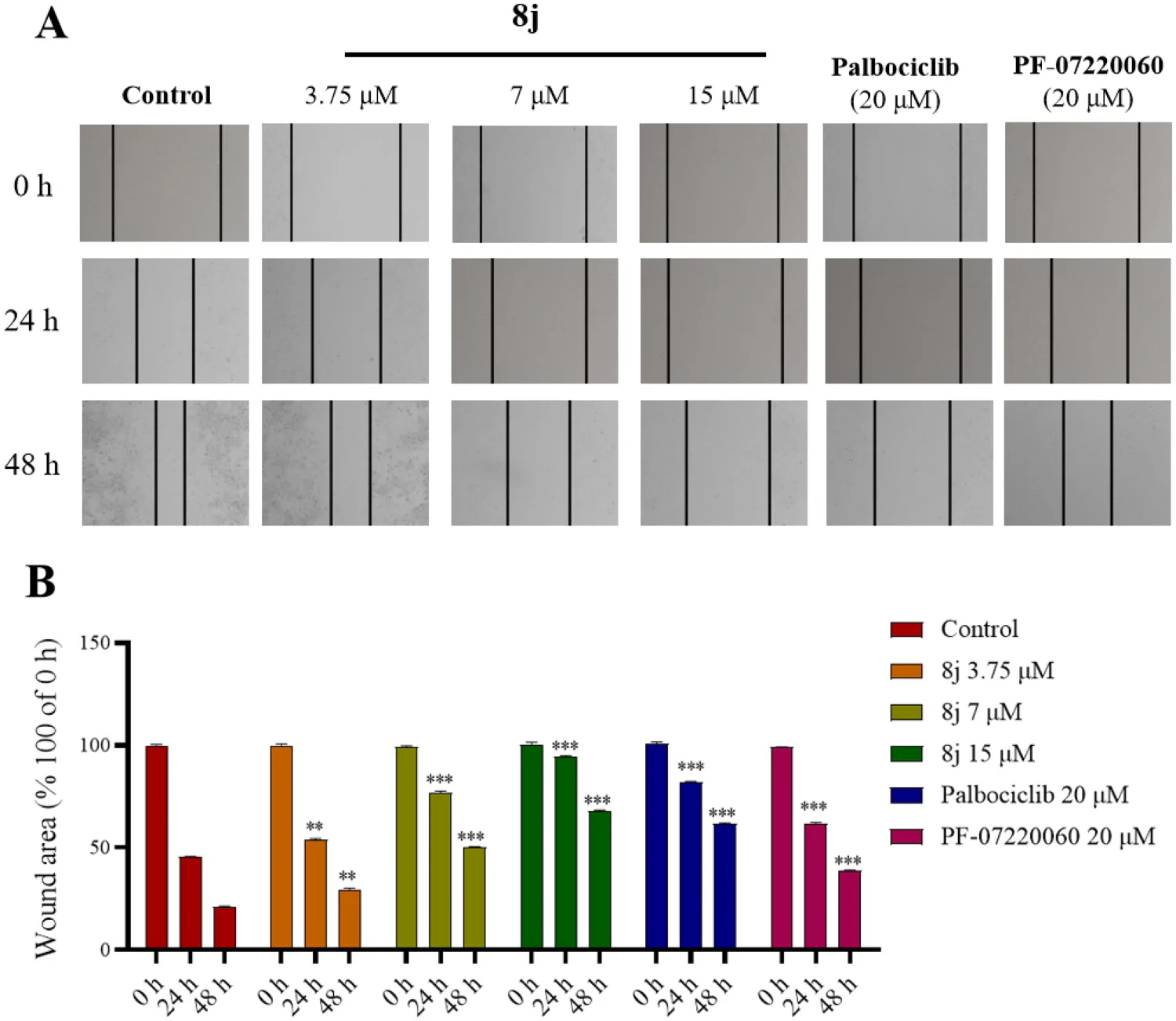
Effects of compound **8j** on the migratory capacity of HCT116 cells. (A) Representative microscopic images of **8j** on HCT116 migration. (B) Quantitative analysis of the relative wound closure rate. **P < 0.01, ***P < 0.001 compared with control for the same time treatment.

### 2.6. Apoptosis induced by 8j

The pro-apoptotic effect of compound **8j** on HCT116 cells was evaluated. Apoptosis was quantified by flow cytometry following Annexin V/propidium iodide dual staining. As depicted in Figure 6A and 6B, exposure to **8j** led to a dose-dependent elevation in the percentage of apoptotic cells relative to the control group. To further examine apoptosis induced by **8j**, Hoechst 33342 staining was performed and analysed by fluorescence microscopy. Control cells displayed intact, homogeneous nuclear morphology (Figure 6C). In contrast, cells treated with **8j** exhibited classical hallmarks of apoptosis, including nuclear fragmentation and chromatin condensation. Together, these data conclusively demonstrate that compound **8j** induces apoptosis in HCT116 cells.

**Figure 6.**
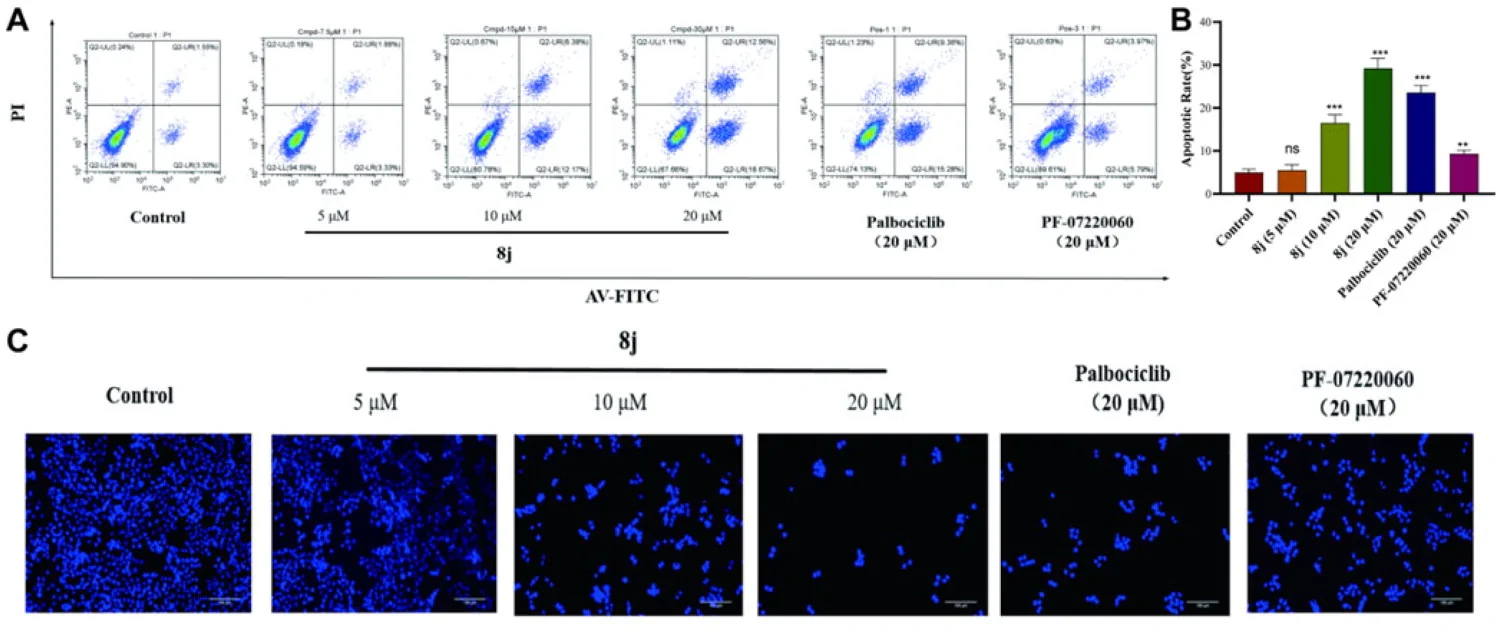
Induction of apoptosis in HCT116 cells by compound **8j**. (A) Representative flow cytometry dot plots of cells stained with Annexin V-FITC and PI after treatment with the indicated concentrations of **8j**. (B) Quantitative analysis of the total apoptotic cell population. **P < 0.01, ***P < 0.001 compared with control. (C) Representative fluorescence micrographs of HCT116 cells stained with Hoechst 33342, showing characteristic apoptotic nuclear morphology following treatment with **8j**.

### 2.7. Cell cycle analysis

To investigate the antiproliferative mechanism of compound **8j**, its effect on cell cycle progression was evaluated. HCT116 cells were treated with the indicated concentrations of **8j**, and the cell cycle distribution was subsequently assessed by flow cytometry following propidium iodide staining. As demonstrated in Figure 7, treatment with **8j** significantly increased the proportion of cells in the G1 phase. These results indicate that compound **8j** effectively induces G0/G1 phase arrest in HCT116 cells, and its mode of action is highly consistent with that of the clinically used CDK4 inhibitors Palbociclib and PF-07220060. This finding provides a mechanistic explanation for the potent anti-proliferative activity of **8j** observed in the colony formation assay: by blocking cell cycle progression, it prevents cells from entering S phase and G2/M phase, thereby fundamentally inhibiting the sustained proliferation capacity of cancer cells. This strongly suggests that compound **8j** may exert its anti-tumour effects by targeting and inhibiting CDK4 kinase activity.

**Figure 7.**
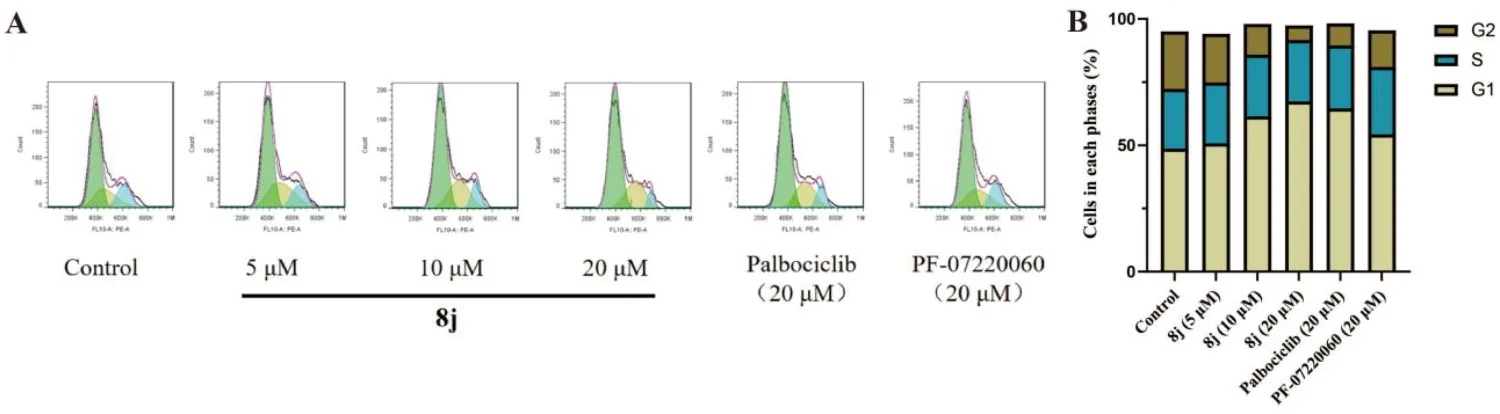
Compound **8j** modulates cell cycle in HCT116 cells. (A) Representative cell cycle profiles of HCT116 cells following 48-h treatment with the indicated concentrations of 8j, as determined by propidium iodide staining and flow cytometric analysis. (B) Quantitative analysis of the percentage of cells in each phase of the cell cycle.

### 2.8. Western blot assay

To elucidate the molecular mechanism responsible for the anti-proliferative activity of compound **8j**, we examined its effects on the expression of key proteins in the CDK4 pathway in HCT116 colon cancer cells using Western blot analysis. As illustrated in Figure 8, treatment with **8j** produced concentration-dependent decrease in the protein levels of CDK4, cyclin D1, and CDK6. At the same concentration (20 μM), **8j** exhibited more potent inhibition of CDK4 and cyclin D1 expression compared to the CDK4 inhibitors Palbociclib and PF-07220060. These results demonstrate that compound **8j** effectively targets and downregulates the key regulators of the G1/S cell cycle transition, providing a plausible molecular basis for its observed growth-inhibitory effects.

**Figure 8.**
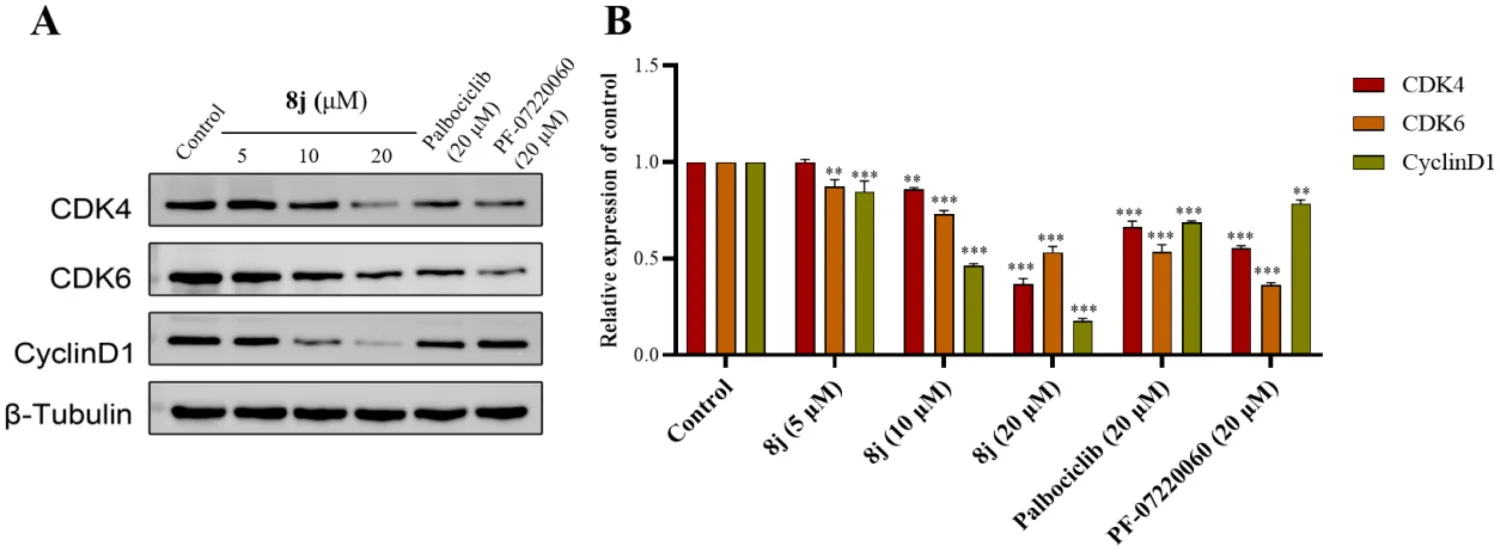
Compound **8j** downregulates the expression of CDK4, Cyclin D1, and CDK6 in HCT116 cells. (A) Representative immunoblots showing protein levels following treatment with the indicated concentrations of **8j**. β-Tubulin served as internal control. (B) Quantitative analysis of relative protein expression levels normalised to β-Tubulin.

## 3. Conclusion

A novel library of benzoxazine-based compounds was designed, synthesised, and evaluated for their potential as CDK4 kinase inhibitors. From this series, compound **8j** was identified as the lead compound, exhibiting potent, broad-spectrum antiproliferative effects *in vitro* against a panel of cancer cell lines. Mechanistic investigations provided compelling evidence that **8j** effectively induced G1 phase cell cycle arrest and promoted apoptosis in HCT116 colon cancer cells, a hallmark of CDK4/6 inhibition. Furthermore, **8j** significantly inhibited colony formation and cell migration, indicating its potential to suppress both proliferation and metastatic capacity. Western blot analysis confirmed that **8j** treatment led to a concentration-dependent downregulation of key cell cycle regulators, including CDK4 and its partner cyclin D1, with potency comparable or superior to the reference inhibitors Palbociclib and PF-07220060. Molecular docking and dynamics simulations substantiated the binding mode of **8j** within the ATP-binding pocket of CDK4, revealing a stable interaction characterised by key hydrogen bonds and hydrophobic contacts, which rationalises its observed biological activity. Collectively, our findings establish compound **8j** as a novel CDK4 inhibitor. The benzoxazine scaffold described herein represents a promising new chemotype for the development of CDK4-targeted therapeutics.

## 4. Experimental section

### 4.1. General chemistry

Reagents and solvents were acquired from commercial suppliers and used as received without further purification unless specified. Reaction progress was monitored via TLC on silica gel plates. Melting points (m.p.) were determined using the XGW X-4B micro-melting point apparatus (Shanghai Yidian Physical Optical Instrument Co., Ltd., China). Purification was carried out by flash column chromatography using silica gel (200–300 mesh). NMR spectra were recorded on a JEOL ECZ 400S spectrometer. HRMS data were obtained in electrospray ionisation (ESI) positive mode on a Waters UPLC-Q–TOF/MS system.

### 4.2. General procedure for the synthesis of compounds 8a–8n

#### 4.2.1. Synthesis of intermediates 3

To a solution of 2-amino-4-bromophenol derivative **1** (18.5 mmol, 1.0 equiv) in dimethyl ethylene glycol (50 ml) was added sodium bicarbonate (4.67 g, 55.6 mmol, 3.0 equiv). The mixture was cooled to 0 °C with stirring, followed by the dropwise addition of chloroacetyl chloride **2** (37 mmol, 2.0 equiv). Upon completion of the addition, the reaction was allowed to warm to room temperature and stirred for 30 min. Caesium carbonate (6.04 g, 18.5 mmol, 1.0 equiv) was then introduced, and the reaction mixture was heated to 80 °C and maintained at this temperature for 1 h. Reaction progress was monitored by liquid chromatography–mass spectrometry. After completion, the mixture was diluted with water and extracted with ethyl acetate. The combined organic extracts were dried over anhydrous sodium sulphate, filtered, and concentrated under reduced pressure to afford the crude intermediate **3**, which was used directly in the subsequent step without further purification.

#### 4.2.2. Synthesis of intermediates 4

Intermediate **3** (18.5 mmol, 1.0 equiv) was dissolved in tetrahydrofuran (30 ml) and cooled to 0 °C. To this solution, borane–tetrahydrofuran complex (37 ml of a 1.0 M solution, 37 mmol, 2.0 equiv) was added dropwise. The reaction mixture was then heated under reflux for 2.5 h, with the reaction progress monitored by TLC. Upon completion, the reaction was carefully quenched by the slow addition of methanol. The mixture was concentrated under reduced pressure, and the resulting crude material was purified by silica gel column chromatography to afford intermediate **4** as a white solid in 43–61% yield.

#### 4.2.3. Synthesis of intermediates 5

To a solution of intermediate **4** (3 mmol, 1.0 equiv) in dry *N*,*N*-dimethylformamide (10 ml) at 0 °C was added sodium hydride (114 mg, 6 mmol, 2.0 equiv). The resulting mixture was stirred for 10 min, after which the appropriate alkylation reagent (4.5 mmol, 1.5 equiv) was added dropwise. The reaction mixture was then allowed to warm to room temperature and stirred for 4 h, with progress monitored by TLC. Upon completion, the reaction was quenched by the careful addition of water. The aqueous mixture was extracted with ethyl acetate, and the combined organic extracts were washed with saturated sodium chloride solution, dried over anhydrous sodium sulphate, and concentrated under reduced pressure. The crude residue was purified by silica gel column chromatography to afford intermediate **5** as a white solid.

#### 4.2.4. Synthesis of intermediates 6

A degassed solution of intermediate **5** (2.7 mmol, 1.0 equiv), bis(pinacolato)diboron (1.38 g, 5.4 mmol, 2.0 equiv), and potassium acetate (535 mg, 5.4 mmol, 2.0 equiv) in 1,4-dioxane (15 ml) was charged with Pd(dppf)Cl_2_ (119 mg, 0.16 mmol, 0.06 equiv). The reaction was conducted under a nitrogen atmosphere with stirring at 90 °C for 6 h. After cooling to room temperature, the mixture was quenched with water and extracted with ethyl acetate. The combined organic extracts were dried over anhydrous sodium sulphate, filtered, and concentrated under reduced pressure to afford the crude intermediate **6**, which was used directly in the subsequent step without further purification.

#### 4.2.5. Synthesis of intermediates 7

A degassed mixture of intermediate **6** (2.7 mmol, 1.0 equiv), 2,4,5-trichloropyrimidine (639 mg, 3.5 mmol, 1.3 equiv), and sodium carbonate (859 mg, 8.1 mmol, 3.0 equiv) in a solvent mixture of 1,4-dioxane (10 ml) and water (3 ml) was treated with tris(triphenylphosphine)palladium (312 mg, 0.27 mmol, 0.1 equiv). The reaction was stirred at 90 °C under a nitrogen atmosphere for 4 h. After cooling to ambient temperature, the mixture was diluted with water and extracted with ethyl acetate. The combined organic extracts were dried over anhydrous sodium sulphate, filtered, and concentrated under reduced pressure. The crude residue was purified by silica gel column chromatography to afford intermediate **7** as a solid in 63–72% yield.

#### 4.2.6. Synthesis of target compound 8

Intermediate **7** (0.6 mmol, 1.0 equiv) and (3S,4R)-4-amino-oxacyclohexan-3-ol hydrochloride (185 mg, 1.2 mmol, 2.0 equiv) were dissolved in acetonitrile (5.0 ml). To this solution, *N,N*-diisopropylethylamine (465 mg, 3.6 mmol, 6.0 equiv) was added. The reaction mixture was stirred at 80 °C for 48 h. After cooling, the solvent was removed under reduced pressure. The residue was diluted with water, basified with a saturated aqueous sodium bicarbonate solution, and extracted with ethyl acetate. The combined organic extracts were dried over anhydrous sodium sulphate, filtered, and concentrated. The crude product was purified by flash column chromatography on silica gel to afford the target compounds **8a**–**8n** as white solids in 52–69% yield.

Characterisation data for compounds (3S,4R)-4-((5-chloro-4-(8-fluoro-4-methyl-3,4- dihydro-2H-benzo[b]^1^^,^^4^oxazin-6-yl)pyrimidin-2-yl)amino)tetrahydro-2H-pyran-3-ol (**8a**) Mp 189–191 °C; ^1^H NMR (400 MHz, Chloroform-*d*): δ 8.26 (s, 1H), 7.04 (dd, *J* = 11.2, 2.0 Hz, 1H), 6.91 (t, *J* = 1.7 Hz, 1H), 5.34 (d, *J* = 5.9 Hz, 1H), 4.43–4.37 (m, 2H), 4.09–3.93 (m, 2H), 3.85–3.77 (m, 1H), 3.61 (td, *J* = 9.5, 4.9 Hz, 1H), 3.44 (td, *J* = 11.8, 2.2 Hz, 1H), 3.37–3.31 (m, 2H), 3.17 (dd, *J* = 11.4, 9.8 Hz, 1H), 2.05–1.99 (m, 1H), 1.76–1.61 (m, 1H).^13^C NMR (101 MHz, Chloroform-*d*): δ 160.73, 158.41, 152.11, 149.71, 138.01, 134.39, 127.75, 117.42, 108.75, 107.07, 72.96, 71.55, 66.61, 65.41, 55.70, 48.63, 39.05, 31.70. HRMS (ESI): exact mass calculated for C_18_H_21_ClFN_4_O_3_ [M + H]^+^, 395.1281; found: 395.1290. Purity, 98.1%.

Characterisation data for compounds (3S,4R)-4-((5-chloro-4-(8-fluoro-4-isopropyl-3,4-dihydro-2H-benzo[b]^1^^,^^4^oxazin-6-yl)pyrimidin-2-yl)amino)tetrahydro-2H-pyran-3-ol (**8b**) Mp 172–174 °C; ^1^H NMR (400 MHz, DMSO-*d*_6_): δ 8.33 (s, 1H), 7.36 (d, *J* = 7.8 Hz, 1H), 7.07–6.91 (m, 2H), 4.91 (d, *J* = 5.4 Hz, 1H), 4.27 (t, *J* = 5.1 Hz, 2H), 4.07 (p, *J* = 6.6 Hz, 1H), 3.83–3.78 (m, 3H), 3.50–3.49 (m, 1H), 3.28 (t, *J* = 4.4 Hz, 2H), 3.02 (t, *J* = 11.1 Hz, 1H), 1.94 (s, 1H), 1.49 (td, *J* = 11.9, 6.1 Hz, 1H), 1.15 (d, *J* = 6.5 Hz, 6H).^13^C NMR (101 MHz, DMSO-*d*_6_): δ 160.60, 158.25, 151.67, 149.30, 136.36, 133.36, 133.23, 128.25, 114.33, 108.74, 104.77, 104.57, 71.37, 68.29, 65.99, 65.01, 54.43, 46.96, 38.02, 31.26, 18.21. HRMS (ESI): exact mass calculated for C_20_H_25_ClFN_4_O_3_ [M + H]^+^, 423.1594; found: 423.1603. Purity, 98.05%.

Characterisation data for compounds (3S,4R)-4-((5-chloro-4-(4-cyclopentyl-8-fluoro −3,4-dihydro-2H-benzo[b]^1^^,^^4^oxazin-6-yl)pyrimidin-2-yl)amino)tetrahydro-2H-pyran-3-ol (**8c**) Mp 160–162 °C; ^1^H NMR (400 MHz, DMSO-*d*_6_): δ 8.33 (s, 1H), 7.36 (d, *J* = 7.9 Hz, 1H), 7.03 (d, *J* = 71.7 Hz, 2H), 4.91 (s, 1H), 4.28 (t, *J* = 5.1 Hz, 2H), 4.18 (p, *J* = 7.4 Hz, 1H), 3.86–3.73 (m, 3H), 3.49 (td, *J* = 9.4, 5.4 Hz, 1H), 3.01 (t, *J* = 10.4 Hz, 1H), 2.05–1.76 (m, 3H), 1.73–1.38 (m, 7H), 1.23 (s, 1H). ^13^C NMR (101 MHz, DMSO-*d*_6_): δ 160.62, 158.46, 158.17, 151.51, 149.14, 137.08, 133.42, 133.30, 128.19, 128.10, 114.32, 109.12, 104.98, 104.78, 71.43, 68.34, 66.04, 65.23, 58.11, 54.48, 31.30, 29.10, 27.55, 23.91. HRMS (ESI): exact mass calculated for C_22_H_27_ClFN_4_O_3_ [M + H]^+^, 449.1750; found: 449.1755. Purity, 99.04%.

Characterisation data for compounds (3S,4R)-4-((5-chloro-4-(8-fluoro-4-(2-methoxyethyl)-3,4-dihydro-2H-benzo[b]^1^^,^^4^oxazin-6-yl)pyrimidin-2-yl)amino)tetrahydro-2H-pyran-3-ol (**8d**) Mp 135–137 °C; ^1^H NMR (400 MHz, DMSO-*d*_6_): δ 8.33 (s, 1H), 7.36 (d, *J* = 7.9 Hz, 1H), 6.98–6.89 (m, 1H), 4.92 (d, *J* = 5.4 Hz, 1H), 4.25 (t, *J* = 5.4 Hz, 2H), 3.87–3.71 (m, 3H), 3.56–3.53 (m, 2H), 3.51–3.39 (m, 5H), 3.26 (s, 3H), 3.03 (t, *J* = 11.1 Hz, 1H), 1.94 (d, *J* = 13.3 Hz, 1H), 1.48 (qd, *J* = 12.0, 4.6 Hz, 1H). ^13^C NMR (101 MHz, DMSO-*d*_6_): δ 160.63, 158.30, 151.59, 149.21, 136.35, 132.88, 132.74, 128.44, 128.35, 114.38, 108.44, 104.84, 104.63, 71.38, 69.11, 68.32, 66.02, 64.48, 58.39, 54.46, 50.19, 47.00, 31.28, 29.10. HRMS (ESI): exact mass calculated for C_20_H_25_ClFN_4_O_4_ [M + H]^+^, 439.1543; found: 439.1551. Purity, 98.06%.

Characterisation data for compounds (3S,4R)-4-((5-chloro-4-(4-(cyclopropylmethyl) −8-fluoro-3,4-dihydro-2H-benzo[b]^1^^,^^4^oxazin-6-yl)pyrimidin-2-yl)amino)tetrahydro-2H-pyran-3-ol (**8e**) Mp 158–160 °C; ^1^H NMR (400 MHz, DMSO-*d*_6_): δ 8.33 (s, 1H), 7.36 (d, *J* = 8.0 Hz, 1H), 6.99 (d, *J* = 49.1 Hz, 2H), 4.91 (d, *J* = 5.4 Hz, 1H), 4.30 (t, *J* = 5.4 Hz, 2H), 3.83–3.75 (m, 3H), 3.58–3.44 (m, 3H), 3.20 (d, *J* = 6.6 Hz, 2H), 3.02 (t, *J* = 11.1 Hz, 1H), 1.94 (d, *J* = 13.1 Hz, 1H), 1.49 (qd, *J* = 12.2, 4.5 Hz, 1H), 1.10–1.02 (m, 1H), 0.64–0.44 (m, 2H), 0.31–0.23 (m, 2H). ^13^C NMR (101 MHz, DMSO-*d*_6_): δ 160.61, 158.19, 151.50, 149.13, 136.75, 133.00, 132.88, 114.30, 108.73, 105.03, 104.83, 71.37, 68.31, 66.00, 64.64, 54.48, 45.90, 31.29, 7.98, 3.30. HRMS (ESI): exact mass calculated for C_21_H_25_ClFN_4_O_3_ [M + H]^+^, 435.1594; found: 435.1605. Purity, 99.29%.

Characterisation data for compounds (3S,4R)-4-((4-(4-benzyl-8-fluoro-3,4-dihydro −2H-benzo[b]^1^^,^^4^oxazin-6-yl)-5-chloropyrimidin-2-yl)amino)tetrahydro-2H-pyran-3-ol (**8f**) Mp 101–103 °C; ^1^H NMR (400 MHz, DMSO-*d*_6_): δ 8.26 (s, 1H), 7.38–7.29 (m, 5H), 7.28-0.20 (m, 1H), 6.94 (s, 2H), 4.87 (d, *J* = 5.4 Hz, 1H), 4.54 (s, 2H), 4.35 (t, *J* = 5.1 Hz, 2H), 3.82–3.71 (m, 3H), 3.54–3.39 (m, 3H), 3.24 (s, 1H), 2.98 (t, *J* = 10.3 Hz, 1H), 1.93–1.83 (m, 1H), 1.43 (qd, *J* = 11.9, 4.5 Hz, 1H). ^13^C NMR (101 MHz, DMSO-*d*_6_): δ 160.57, 158.31, 151.58, 149.20, 137.85, 136.57, 132.93, 132.80, 128.67, 128.36, 128.27, 126.98, 114.25, 108.90, 105.22, 105.01, 71.29, 68.30, 65.93, 64.64, 54.34, 54.30, 46.87, 31.22. HRMS (ESI): exact mass calculated for C_24_H_25_ClFN_4_O_3_ [M + H]^+^, 471.1594; found: 471.1609. Purity, 99.39%.

Characterisation data for compounds 1-(6-(5-chloro-2-(((3S,4R)-3-hydroxytetrahydro −2H-pyran-4-yl)amino)pyrimidin-4-yl)-8-fluoro-2,3-dihydro-4H-benzo[b]^1^^,^^4^oxazin-4-yl)ethan-1-one (**8 g**) Mp 116–118 °C; ^1^H NMR (400 MHz, DMSO-*d*_6_): δ 8.37 (s, 1H), 7.70–6.94 (m, 2H), 4.90 (d, *J* = 5.4 Hz, 1H), 4.41 (t, *J* = 4.5 Hz, 2H), 3.94 (t, *J* = 5.4 Hz, 2H), 3.84–3.76 (m, 3H), 3.51–3.44 (m, 1H), 3.03 (dd, *J* = 11.1, 9.7 Hz, 1H), 2.30 (s, 3H), 1.93 (s, 1H), 1.55–1.40 (m, 1H). ^13^C NMR (101 MHz, DMSO-*d*_6_): δ 169.07, 160.68, 158.36, 151.55, 149.15, 136.54, 127.88, 126.89, 120.44, 114.30, 112.14, 111.96, 71.32, 68.33, 66.58, 65.99, 54.34, 31.24, 23.21. HRMS (ESI): exact mass calculated for C_19_H_20_ClFN_4_O_4_Na [M + Na]^+^, 445.1049; found: 445.1067. Purity, 99.54%.

Characterisation data for compounds (6-(5-chloro-2-(((3S,4R)-3-hydroxytetrahydro −2H-pyran-4-yl)amino)pyrimidin-4-yl)-8-fluoro-2,3-dihydro-4H-benzo[b]^1^^,^^4^oxazin-4-yl)(cyclopropyl)methanone (**8h**) Mp 118–120 °C; ^1^H NMR (400 MHz, DMSO-*d*_6_): δ 8.37 (s, 1H), 8.02 (s, 1H), 7.49–7.39 (m, 2H), 4.90 (d, *J* = 5.4 Hz, 1H), 4.42 (t, *J* = 4.7 Hz, 2H), 4.05 (s, 2H), 3.82–3.76 (m, 3H), 3.48 (m, 1H), 3.07–2.97 (m, 1H), 2.20–2.15 (m, 1H), 1.92 (d, *J* = 13.2 Hz, 1H), 1.48 (qd, *J* = 11.8, 4.5 Hz, 1H), 1.13–0.72 (m, 4H). ^13^C NMR (101 MHz, DMSO-*d*_6_): δ 171.56, 160.70, 158.67, 151.72, 149.31, 136.58, 136.45, 127.79, 126.89, 120.21, 114.22, 112.33, 112.13, 71.33, 68.31, 66.99, 65.98, 54.30, 31.26, 13.10, 8.87. HRMS (ESI): exact mass calculated for C_21_H_22_ClFN_4_O_4_Na [M + Na]^+^, 471.1206; found: 471.1233. Purity, 99.08%.

Characterisation data for compounds (3S,4R)-4-((5-chloro-4-(8-fluoro-3,4-dihydro −2H-benzo[b]^1^^,^^4^oxazin-6-yl)pyrimidin-2-yl)amino)tetrahydro-2H-pyran-3-ol (**8i**) Mp 111–113 °C; ^1^H NMR (400 MHz, DMSO-*d*_6_): δ 8.32 (s, 1H), 7.34 (d, *J* = 8.0 Hz, 1H), 6.89 (s, 1H), 6.82 (d, *J* = 11.7 Hz, 1H), 6.32 (s, 1H), 4.90 (d, *J* = 5.2 Hz, 1H), 4.22 (t, *J* = 5.0, 2H), 3.85–3.72 (m, 3H), 3.47 (m, 1H), 3.03 (t, *J* = 11.1 Hz, 1H), 2.03–1.81 (m, 1H), 1.59–1.37 (m, 1H). ^13^C NMR (101 MHz, DMSO-*d*_6_): δ 160.66, 158.23, 151.62, 149.24, 136.54, 136.50, 132.24, 132.11, 128.38, 114.26, 110.96, 104.51, 104.31, 71.31, 68.34, 65.97, 65.02, 54.25, 31.27. HRMS (ESI): exact mass calculated for C_17_H_19_ClFN_4_O_3_ [M + H]^+^, 381.1124; found: 381.1129. Purity, 98.10%.

Characterisation data for compounds (3S,4R)-4-((5-chloro-4-(8-fluoro-4-isopropyl −2,2-dimethyl-3,4-dihydro-2H-benzo[b]^1^^,^^4^oxazin-6-yl)pyrimidin-2-yl)amino)tetrahydro-2H-pyran-3-ol (**8j**) Mp 152–154 °C; ^1^H NMR (400 MHz, DMSO-*d*_6_): δ 8.33 (s, 1H), 7.35 (d, *J* = 7.9 Hz, 1H), 7.09–6.95 (m, 2H), 4.91 (d, *J* = 5.4 Hz, 1H), 4.12 (p, *J* = 6.6 Hz, 1H), 3.95–3.68 (m, 4H), 3.66–3.41 (m, 1H), 3.02 (t, *J* = 2.7 Hz, 3H), 1.94 (s, 1H), 1.56–1.42 (m, 1H), 1.31 (s, 6H), 1.16 (d, *J* = 6.5 Hz, 6H). ^13^C NMR (101 MHz, DMSO-*d*_6_): δ 160.60, 158.00, 152.00, 149.63, 134.97, 127.68, 114.30, 107.84, 104.94, 104.73, 73.61, 71.38, 68.31, 66.00, 54.43, 46.87, 46.16, 31.28, 29.08, 24.94, 18.49, 18.46. HRMS (ESI): exact mass calculated for C_22_H_29_ClFN_4_O_3_ [M + H]^+^, 451.1907; found: 451.1918. Purity, 98.18%.

Characterisation data for compounds 6-(5-chloro-2-(((3S,4R)-3-hydroxytetrahydro −2H-pyran-4-yl)amino)pyrimidin-4-yl)-8-fluoro-4-isopropyl-2H-benzo[b]^1^^,^^4^oxazin-3(4H)-one (**8k**) Mp 109–111 °C; ^1^H NMR (400 MHz, DMSO-*d*_6_): δ 8.40 (s, 1H), 7.48–7.39 (m, 3H), 4.91 (d, *J* = 5.4 Hz, 1H), 4.73 (s, 3H), 3.86–3.77 (m, 3H), 3.53–3.46 (m, 1H), 3.27 (s, 1H), 3.01 (t, *J* = 10.4 Hz, 1H), 1.96–1.92 (m, 1H), 1.48 (d, *J* = 6.9 Hz, 7H). ^13^C NMR (101 MHz, DMSO-*d*_6_): δ 164.98, 160.66, 158.82, 151.64, 149.23, 130.70, 130.34, 130.26, 114.26, 111.80, 111.60, 71.40, 68.31, 68.06, 66.02, 54.43, 31.24, 19.51. HRMS (ESI): exact mass calculated for C_20_H_23_ClFN_4_O_4_ [M + H]^+^, 437.1386; found: 437.1386. Purity, 98.13%.

Characterisation data for compounds (3S,4R)-4-((5-chloro-4-(4-isopropyl-3,4-dihydro −2H-benzo[b]^1^^,^^4^oxazin-6-yl)pyrimidin-2-yl)amino)tetrahydro-2H-pyran-3-ol (**8 l**) Mp 137–139 °C; ^1^H NMR (400 MHz, DMSO-*d*_6_): δ 8.31 (s, 1H), 7.44–7.14 (m, 2H), 7.02 (s, 1H), 6.77 (d, *J* = 8.2 Hz, 1H), 4.92 (s, 1H), 4.45–4.08 (m, 2H), 4.06 (p, *J* = 6.5 Hz, 1H), 3.82–3.77 (m, 3H), 3.50 (s, 1H), 3.26–3.13 (m, 2H), 3.08–2.95 (m, 1H), 1.95 (s, 1H), 1.48 (qd, *J* = 12.1, 4.4 Hz, 1H), 1.15 (d, *J* = 6.6 Hz, 6H). ^13^C NMR (101 MHz, DMSO-*d*_6_): δ 160.65, 157.77, 145.64, 134.54, 129.26, 118.01, 115.42, 114.40, 113.12, 71.41, 68.32, 66.03, 64.94, 54.44, 46.38, 38.15, 31.31, 29.09, 18.11, 18.07. HRMS (ESI): exact mass calculated for C_20_H_26_ClN_4_O_3_ [M + H]^+^, 405.1688; found: 405.1703. Purity, 98.91%.

Characterisation data for compounds (3S,4R)-4-((5-chloro-4-(4-isopropyl-8-methoxy −3,4-dihydro-2H-benzo[b]^1^^,^^4^oxazin-6-yl)pyrimidin-2-yl)amino)tetrahydro-2H-pyran-3-ol (**8 m**) Mp 94–96 °C; ^1^H NMR (400 MHz, DMSO-*d*_6_): δ 8.32 (s, 1H), 7.32 (d, *J* = 7.8 Hz, 1H), 6.86 (d, *J* = 68.2 Hz, 2H), 4.92 (s, 1H), 4.19 (t, *J* = 5.2, 3.5 Hz, 2H), 4.04 (p, *J* = 6.6 Hz, 1H), 3.85 − 3.75 (m, 3H), 3.73 (s, 3H), 3.53 (s, 1H), 3.21 (t, 2H), 3.02 (t, *J* = 10.4 Hz, 1H), 1.95 (s, 1H), 1.50 (td, *J* = 12.1, 4.3 Hz, 1H), 1.13 (d, *J* = 6.6 Hz, 7H). ^13^C NMR (101 MHz, DMSO-*d*_6_): δ 160.61, 158.15, 147.67, 134.91, 128.06, 114.45, 107.04, 102.56, 71.43, 68.31, 66.03, 64.65, 55.72, 54.50, 46.89, 38.18, 31.31, 29.12, 18.26, 18.23. HRMS (ESI): exact mass calculated for C_21_H_28_ClN_4_O_4_ [M + H]^+^, 435.1794; found: 435.1801. Purity, 98.02%.

Characterisation data for compounds (3S,4R)-4-((5-chloro-4-(7-fluoro-4-isopropyl −3,4-dihydro-2H-benzo[b]^1^^,^^4^oxazin-6-yl)pyrimidin-2-yl)amino)tetrahydro-2H-pyran-3-ol (**8n**) Mp 85–87 °C; ^1^H NMR (400 MHz, DMSO-*d*_6_): δ 8.35 (s, 1H), 7.42 (s, 1H), 6.78 (d, *J* = 7.0 Hz, 1H), 6.68 (d, *J* = 10.7 Hz, 1H), 4.90 (d, *J* = 5.2 Hz, 1H), 4.24 (t, *J* = 4.4 Hz, 2H), 4.00 (p, *J* = 6.6 Hz, 1H), 3.84–3.76 (m, 4H), 3.48 (s, 0H), 3.18 (s, 2H), 3.02 (t, *J* = 10.6 Hz, 1H), 1.94 (s, 1H), 1.46 (qd, *J* = 11.9, 4.6 Hz, 1H), 1.10 (t, *J* = 6.6, 4.8 Hz, 6H). ^13^C NMR (101 MHz, DMSO-*d*_6_): δ 160.69, 156.89, 152.05, 149.68, 146.04, 131.78, 116.68, 116.48, 113.09, 103.73, 103.47, 71.36, 68.26, 65.97, 65.47, 54.36, 46.59, 37.88, 31.22, 18.03, 17.77. HRMS (ESI): exact mass calculated for C_20_H_25_ClFN_4_O_3_ [M + H]^+^, 423.1594; found: 423.1613. Purity, 97.94%.

### 4.2. Biological evaluation

#### 4.2.1. Cell lines and cell culture

Four human cancer cell lines, including MDA-MB-231 (breast) and MCF-7 (breast), HCT-116 (colorectal carcinoma), and HepG2 (hepatocellular carcinoma), were utilised in this study. All cell lines were acquired from the Cell Bank of the Shanghai Institute of Biochemistry and Cell Biology. Cells were routinely maintained in a humidified atmosphere of 5% CO_2_ at 37 °C. Culture media consisted of either high-glucose Dulbecco’s Modified Eagle Medium or RPMI-1640, each supplemented with 10% foetal bovine serum and 1% (v/v) antibiotic-antimycotic solution containing 100 units/mL penicillin G sodium and 100 μg/ml streptomycin.

#### 4.2.2. MTT assay

Cell viability was assessed using the MTT assay. After seeding in 96-well plates at 3 × 10^3^ cells per well and overnight adherence, cells were treated with 0.5% DMSO or specified compound concentrations for 72 h. Subsequently, 20 μL of MTT solution was added to each well, followed by a 4 h incubation at room temperature. The resulting formazan crystals were then solubilised by adding 150 μL of DMSO to each well. Absorbance was recorded at a wavelength of 570 nm using a microplate reader. The IC_50_ for each compound was determined by GraphPad Prism software.

#### 4.2.3. Molecular docking and molecular dynamics studies

The X-ray crystal structure of CDK4 (PDB ID: 9PE8) was retrieved from the Protein Data Bank. The chemical structure of compound **8j** was sketched using ChemDraw and converted into SDF format. Both protein and ligand files were prepared by adding polar hydrogen atoms and converted into PDBQT format. Molecular docking was performed using AutoDock Vina 1.2.2. The docking grid box was centred at coordinates (14.302, 27.538, 8.063) with dimensions of 17.6 Å × 17.6 Å × 17.6 Å and a grid spacing of 0.05 nm. The binding affinity and the optimal binding pose were determined based on the lowest binding energy. Systematic analysis of the protein–ligand interface was conducted using PLIP and LigPlus, with final visualisations generated using PyMOL 2.5.

The thermodynamic stability and conformational dynamics of the CDK4-ligand complex were evaluated through molecular dynamics simulations. The initial system was constructed by parameterising the CDK4 protein with the CHARMM36 force field, while ligand topology was prepared using the generalised amber force field. The solute was immersed in a periodic cubic simulation box containing TIP3P water molecules, with a stipulated minimum solute-to-wall distance of 1.0 nm. System charge was neutralised by adding appropriate numbers of Na^+^ and Cl^-^ ions. Energy minimisation was carried out employing steepest descent and conjugate gradient algorithms. The system was subsequently equilibrated in two phases: first under the NVT ensemble for 100 ps, followed by the NPT ensemble for 100 ps, maintaining a temperature of 300 K and a pressure of 1 bar. Production dynamics were then conducted for 100 ns with an integration time step of 2 fs, using the Particle Mesh Ewald method for long-range electrostatic interactions. All simulations were performed using GROMACS 2024.5. Conformational sampling was further analysed via principal component analysis, and the associated Gibbs free energy landscape was calculated to assess thermodynamic stability.

#### 4.2.4. Clonogenic assay

The clonogenic potential of HCT116 cells was assessed using a colony formation assay. HCT116 cells were seeded in 6-well plates at 3 × 10^3^ cells per well. After overnight incubation to allow for cell attachment, the medium was replaced with fresh medium containing the test compounds. Cells were treated with compound 8j at final concentrations of 5, 10, and 20 µM. For comparison, parallel treatments with the reference compounds Palbociclib and PF-07220060, each at 20 µM, were included. A vehicle control group, containing an equivalent volume of DMSO, was used as the untreated control. Fresh culture medium containing these compounds was changed every three days until cell colonies are visible. After about 14 days later, the culture medium was removed, the cells were then fixed with methanol and stained with a 0.1% (w/v) crystal violet solution. Colonies were visualised and digitally captured. For quantification, the bound crystal violet was extracted with 30% glacial acetic acid, and the optical density at 570 nm was subsequently determined using a microplate reader. All assays were performed in at least three independent replicates.

#### 4.2.5. Cell migration assay

Cell migration was evaluated using a standardised wound healing assay. A confluent monolayer of HCT116 cells was established in 6-well plates. A uniform linear wound was then generated in the monolayer using a sterile 200 μL pipette tip. Cells were then exposed to serum-free medium containing specified concentrations of the test compounds: **8j** (3.75, 7, and 15 μM), Palbociclib (20 μM), or PF-07220060 (20 μM). Digital images of the wound area were captured at 0, 24, and 48 h post-treatment. The migratory capacity was assessed by quantifying the residual wound area using ImageJ software. Each experimental condition was performed in at least three independent replicates.

#### 4.2.6. Assessment of apoptosis

Apoptosis was assessed by flow cytometry in accordance with the manufacturer’s protocol using an Annexin V-FITC/PI detection kit. HCT116 cells were treated for 48 h with 0.5% DMSO or graded concentrations of compound **8j**, Palbociclib, or PF-07220060. After treatment, cells were harvested, subjected to two washes with phosphate-buffered saline, and subsequently stained with 5 μL of propidium iodide and 5 μL of Annexin V-FITC under light-protected conditions. Samples were analysed on a BD FACSVerse flow cytometer, and the percentages of early and late apoptotic cells were determined using BD FACSuite software.

##### 4.2.7. Hoechst 33342 staining assay

HCT116 cells were plated in 6-well plates at 3 × 10^5^ cells per well, followed by treatment with the specified pharmacological agents. Upon completion of the treatment period, cells were washed twice with PBS. Nuclear staining was then conducted by incubating the cells with 1 ml of Hoechst 33342 (10 μg/mL) per well for 15 min in a humidified incubator. Following two additional PBS washes, 1 ml of fresh PBS was added to each well. Cells were examined under a fluorescence microscope to assess morphological features indicative of apoptosis, and representative images were captured.

#### 4.2.8. Cell cycle analysis

Under the indicated treatment conditions, HCT116 cells were maintained for 48 h. Following incubation, cells were collected and fixed in 70% ethanol on ice. Nuclear DNA was stained with propidium iodide according to the manufacturer’s protocol. Data acquisition was carried out using flow cytometry.

#### 4.2.9. Western blot analysis

HCT116 cells were treated for 48 h with 0.5% DMSO or indicated concentrations of compound **8j**, Palbociclib, or PF-07220060. Total protein was subsequently extracted by lysing the harvested cells with RIPA buffer. Protein samples were subsequently resolved by electrophoresis on 10% SDS-polyacrylamide gels and electrotransferred onto PVDF membranes (Millipore). Membranes were incubated with 5% non-fat dry milk for 1 h before incubation with specific primary antibodies. Following overnight incubation with primary antibodies targeting CDK4, Cyclin D1, and CDK6 at 4 °C, the membranes were washed three times with TBST. Subsequently, they were incubated with an HRP-conjugated secondary antibody for 1 h. Immunoreactive bands were detected using an enhanced chemiluminescence (ECL) system, and band intensity was quantified with ImageJ software to determine relative protein expression levels.

## Data Availability

The data that support the findings of this study are available from the corresponding author upon reasonable request.
